# Supplementary material for: Photosymbiosis shaped animal genome architecture and gene evolution as revealed in giant clams
Source: Commun Biol. 2025 Jan 4;8:7. doi: 10.1038/s42003-024-07423-8 (PMC11700175; doi:10.1038/s42003-024-07423-8)
Supplement: Supplementary file 1 — Supplementary Materials [file 42003_2024_7423_MOESM1_ESM.pdf]

Supplementary Materials

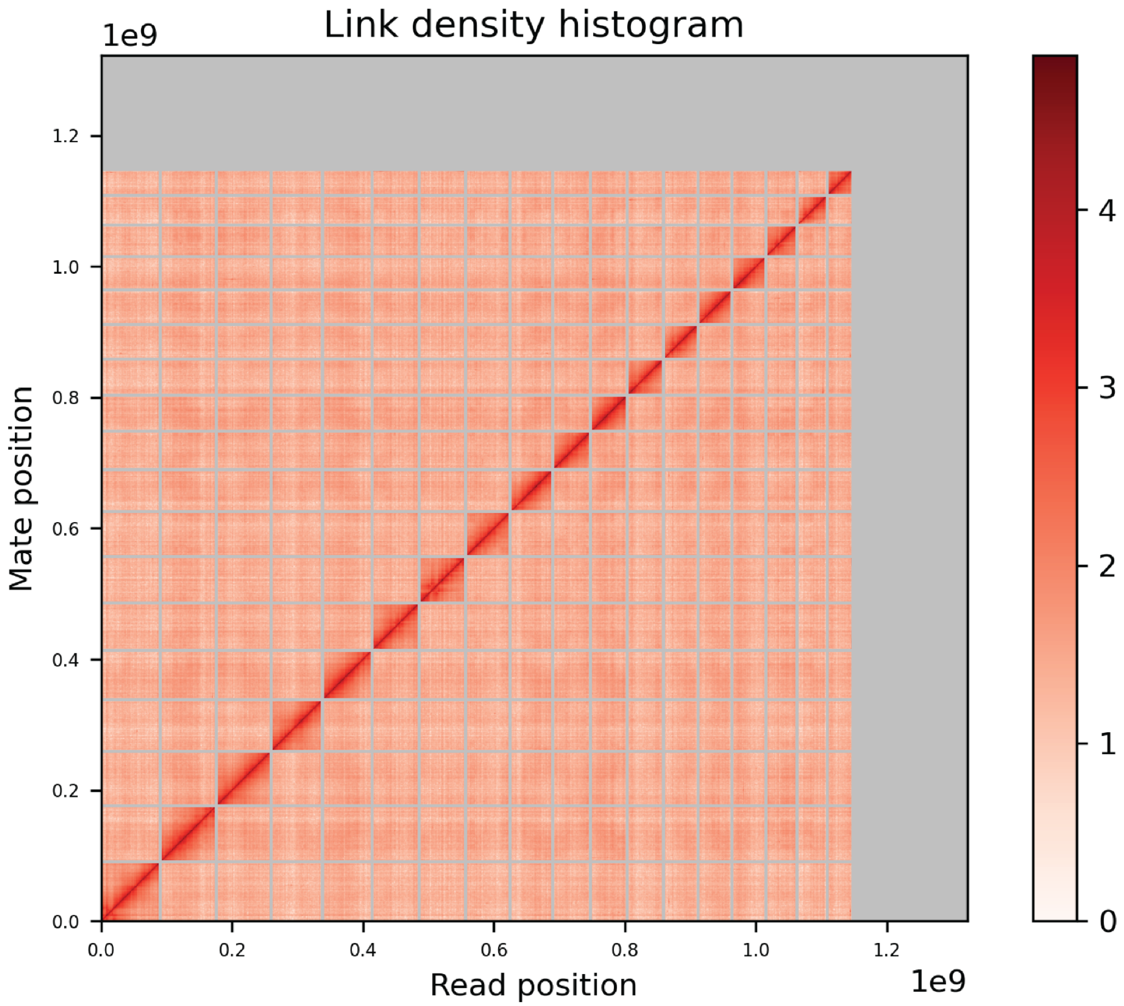

Supplementary Figure 1. Omni-C linkage heatmap depicting the genome-wide analysis of chromatin interactions in the *Tridacna maxima* genome, revealing the organization into 18 pseudo-chromosomes.
