# Supplementary material for: Photosymbiosis shaped animal genome architecture and gene evolution as revealed in giant clams
Source: Commun Biol. 2025 Jan 4;8:7. doi: 10.1038/s42003-024-07423-8 (PMC11700175; doi:10.1038/s42003-024-07423-8)
Supplement: Supplementary file 2 — Description of Additional Supplementary Files [file 42003_2024_7423_MOESM2_ESM.pdf]

Description of Additional Supplementary Files

File name: Supplementary Data 1
Description: Selected enriched GO terms from taxon-specific genes and gene families that are unique to the three Tridacna species, compared to thirteen other molluscan species, indicating potential functions related to the symbiotic lifestyle. It's worth noting that many of these enriched GO terms overlap with those found in Tridacna maxima, as highlighted in Table 1.

File name: Supplementary Data 2
Description: Assessment of Benchmarking Universal Single-Copy Orthologs (BUSCO) completeness for the raw assembly, post-RNA-Seq re-annotation using NCBI data, and after manual curation, with an appendix on manual BLAST results for missing BUSCOs.

File name: Supplementary Data 3
Description: Comprehensive list of 122 expanded gene families in Tridacna maxima compared to thirteen other molluscan genomes.

File name: Supplementary Data 4
Description: Comprehensive list of 85 contracted gene families in Tridacna maxima compared to thirteen other molluscan genomes.

File name: Supplementary Data 5
Description: Comprehensive list of 39 expanded gene families in three Tridacna species compared to thirteen other molluscan genomes, identified using the OrthoFinder pipeline. Only gene families containing more than 1 gene in all three Tridacna species are selected.

File name: Supplementary Data 6
Description: Comprehensive list of 39 contracted gene families in three Tridacna species compared to thirteen other molluscan genomes, identified using the OrthoDB pipeline.

File name: Supplementary Data 7: Summary of functions of 5742 novel genes in Tridacna maxima. These genes are unique to the species and not assigned to any gene families.

File name: Supplementary Data 8
Description: Summary of functions of 1756 gene families (9077 genes) unique to Tridacna maxima. These gene families were not identified in the other thirteen molluscan genomes.

File name: Supplementary Data 9
Description: Full list of significantly enriched GO terms from genes and gene families unique to Tridacna maxima, identified using GO_MWU.

File name: Supplementary Data 10
Description: Summary of functions of 419 gene families unique to the three Tridacna species, and also shared by all three species.

File name: Supplementary Data 11
Description: Full list of significantly enriched GO terms from genes and gene families unique to the three Tridacna species, identified using GO_MWU.

File name: Supplementary Data 12
Description: Detailed list of the 48 RNA-Seq libraries obtained from NCBI used for the re-annotation of the Tridacna maxima genome.
