# Supplementary material for: Photosymbiosis shaped animal genome architecture and gene evolution as revealed in giant clams
Source: Commun Biol. 2025 Jan 4;8:7. doi: 10.1038/s42003-024-07423-8 (PMC11700175; doi:10.1038/s42003-024-07423-8)
Supplement: Supplementary file 4 — Reporting summary [file 42003_2024_7423_MOESM4_ESM.pdf]

nature portfolio

Corresponding author(s): Ruiqi Li

Last updated by author(s): Dec 10, 2024

Reporting Summary

Nature Portfolio wishes to improve the reproducibility of the work that we publish. This form provides structure for consistency and transparency in reporting. For further information on Nature Portfolio policies, see our Editorial Policies and the Editorial Policy Checklist.

Statistics

For all statistical analyses, confirm that the following items are present in the figure legend, table legend, main text, or Methods section.

n/a	Confirmed	
☒	☐	The exact sample size (*n*) for each experimental group/condition, given as a discrete number and unit of measurement
☒	☐	A statement on whether measurements were taken from distinct samples or whether the same sample was measured repeatedly
☒	☐	The statistical test(s) used AND whether they are one- or two-sided<br>*Only common tests should be described solely by name; describe more complex techniques in the Methods section.*
☒	☐	A description of all covariates tested
☒	☐	A description of any assumptions or corrections, such as tests of normality and adjustment for multiple comparisons
☒	☐	A full description of the statistical parameters including central tendency (e.g. means) or other basic estimates (e.g. regression coefficient) AND variation (e.g. standard deviation) or associated estimates of uncertainty (e.g. confidence intervals)
☒	☐	For null hypothesis testing, the test statistic (e.g. *F*, *t*, *r*) with confidence intervals, effect sizes, degrees of freedom and *P* value noted<br>*Give P values as exact values whenever suitable.*
☒	☐	For Bayesian analysis, information on the choice of priors and Markov chain Monte Carlo settings
☒	☐	For hierarchical and complex designs, identification of the appropriate level for tests and full reporting of outcomes
☒	☐	Estimates of effect sizes (e.g. Cohen's *d*, Pearson's *r*), indicating how they were calculated

Our web collection on statistics for biologists contains articles on many of the points above.

Software and code

Policy information about availability of computer code

Data collection	No software was used for data collection
Data analysis	Please see the details in the method session:<br>Wtdbg2, blobtools v1.1.1 , Purge_dups v1.2.3 , Dovetail HiRise Scaffolding pipeline, Trinity v.2.9.1, Trimmomatic v.0.35, RepeatModeler v.2.0.1, RepeatMasker 4.1.0, MAKER v.3.01.03, Augustus v.3.3.3, sratoolkit v.3.0.0, STAR v.2.7.4, StringTie v.2.2.1, TransDecoder v.5.6.0, BLASTP v.2.10, hmmscan v.3.3, BUSCO v.5.4.2, circos v0.69.9, qualimap v.2.2.1, samtools, PSMC, MCScanX, PANNZER2, TrimAL v1.4, mafft v7.508, RAxML v8.2.12, MEGAX v10.2.4, OrthoFinder

For manuscripts utilizing custom algorithms or software that are central to the research but not yet described in published literature, software must be made available to editors and reviewers. We strongly encourage code deposition in a community repository (e.g. GitHub). See the Nature Portfolio guidelines for submitting code & software for further information.

Data

Policy information about availability of data

All manuscripts must include a data availability statement. This statement should provide the following information, where applicable:

- Accession codes, unique identifiers, or web links for publicly available datasets
- A description of any restrictions on data availability
- For clinical datasets or third party data, please ensure that the statement adheres to our policy

The sequencing data generated for this study can be accessed through the National Center for Biotechnology Information (NCBI) Sequence Read Archive (SRA) under the BioProject PRJNA1077608. The genome assembly and annotation are deposited in NCBI Genome Portal under accession number JBAJIZ000000000.

Research involving human participants, their data, or biological material

Policy information about studies with human participants or human data. See also policy information about sex, gender (identity/presentation), and sexual orientation and race, ethnicity and racism.

Reporting on sex and gender	No human participants were involved in this study
Reporting on race, ethnicity, or other socially relevant groupings	No human participants were involved in this study
Population characteristics	No human participants were involved in this study
Recruitment	No human participants were involved in this study
Ethics oversight	No human participants were involved in this study

Note that full information on the approval of the study protocol must also be provided in the manuscript.

Field-specific reporting

Please select the one below that is the best fit for your research. If you are not sure, read the appropriate sections before making your selection.

☒ Life sciences ☐ Behavioural & social sciences ☐ Ecological, evolutionary & environmental sciences

For a reference copy of the document with all sections, see nature.com/documents/nr-reporting-summary-flat.pdf

Life sciences study design

All studies must disclose on these points even when the disclosure is negative.

Sample size	We sequenced the whole genome from one individual.
Data exclusions	Low-quality reads and adapter sequences were removed from the raw data.
Replication	One specimen from the species was used, and the identification was confirmed by its morphological features.
Randomization	Not applicable.
Blinding	Not applicable.

Reporting for specific materials, systems and methods

We require information from authors about some types of materials, experimental systems and methods used in many studies. Here, indicate whether each material, system or method listed is relevant to your study. If you are not sure if a list item applies to your research, read the appropriate section before selecting a response.

Materials & experimental systems

n/a	Involved in the study
☒	☐ Antibodies
☒	☐ Eukaryotic cell lines
☒	☐ Palaeontology and archaeology
☐	☒ Animals and other organisms
☒	☐ Clinical data
☒	☐ Dual use research of concern
☒	☐ Plants

Methods

n/a	Involved in the study
☒	☐ ChIP-seq
☒	☐ Flow cytometry
☒	☐ MRI-based neuroimaging

Animals and other research organisms

Policy information about studies involving animals; ARRIVE guidelines recommended for reporting animal research, and Sex and Gender in Research

Laboratory animals	This study did not use laboratory animals.
Wild animals	A live adult specimen of Tridacna maxima was collected from Luminao reef flat in Guam at 1 meter depth under collection permit SC-MPA-20-003, then shipped to the USA for library construction and sequencing (Export permit: CO-21-010).
Reporting on sex	Giant clams are hermaphrodites.
Field-collected samples	The specimen was dissected immediately after collecting.
Ethics oversight	This study did not require ethic approval

Note that full information on the approval of the study protocol must also be provided in the manuscript.

Plants

Seed stocks	NA
Novel plant genotypes	NA
Authentication	NA
